# Assessment of Uptake Appropriateness of Computed Tomography for Lung Cancer Screening According to Patients Meeting Eligibility Criteria of the US Preventive Services Task Force

**DOI:** 10.1001/jamanetworkopen.2022.43163

**Published:** 2022-11-21

**Authors:** Yu Liu, I-Wen Elaine Pan, Hyo Jung Tak, Ioannis Vlahos, Robert Volk, Ya-Chen Tina Shih

**Affiliations:** 1 Department of Health Services Research, The University of Texas MD Anderson Cancer Center, Houston; 2 Department of Health Services Research and Administration, University of Nebraska Medical Center, Omaha; 3 Thoracic Imaging Department, The University of Texas MD Anderson Cancer Center, Houston

## Abstract

**Importance:**

Currently, computed tomography (CT) is used for lung cancer screening (LCS) among populations with various levels of compliance to the eligibility criteria from the US Preventive Services Task Force (USPSTF) recommendations and may represent suboptimal allocation of health care resources.

**Objective:**

To evaluate the appropriateness of CT LCS according to the USPSTF eligibility criteria.

**Design, Setting, and Participants:**

This cross-sectional study used the 2019 Behavioral Risk Factor Surveillance System (BRFSS) survey. Participants included individuals who responded to the LCS module administered in 20 states and had valid answers to questions regarding screening and smoking history. Data were analyzed between October 2021 and August 2022.

**Exposures:**

Screening eligibility groups were categorized according to the USPSTF 2013 recommendations, and subgroups of individuals who underwent LCS were analyzed.

**Main Outcomes and Measures:**

Main outcomes included LCS among the screening-eligible population and the proportions of the screened populations according to compliance categories established from the USPSTF 2013 and 2021 recommendations. In addition, the association between respondents’ characteristics and LCS was evaluated for the subgroup who were screened despite not meeting any of the 3 USPSTF screening criteria: age, pack-year, and years since quitting smoking.

**Results:**

A total of 96 097 respondents were identified for the full study cohort, and 2 subgroups were constructed: (1) 3374 respondents who reported having a CT or computerized axial tomography to check for lung cancer and (2) 33 809 respondents who did not meet any screening eligibility criteria. The proportion of participants who were under 50 years old was 53.1%; between 50 and 54, 9.1%; between 55 and 79, 33.8%; and over 80, 4.0%. A total of 51 536 (50.9%) of the participants were female. According to the USPSTF 2013 recommendation, 807 (12.8%) of the screening-eligible population underwent LCS. Among those who were screened, only 807 (20.9%) met all 3 screening eligibility criteria, whereas 538 (20.1%) failed to meet any criteria. Among respondents in subgroup 2, being of older age and having a history of stroke, chronic obstructive pulmonary disease, kidney disease, or diabetes were associated with higher likelihood of LCS.

**Conclusions and Relevance:**

In this cross-sectional study of the BRFSS 2019 survey, the low uptake rate among screening-eligible patients undermined the goal of LCS of early detection. Suboptimal screening patterns could increase health system costs and add financial stress, psychological burden, and physical harms to low-risk patients, while failing to provide high-quality preventive services to individuals at high risk of lung cancer.

## Key Points

**Question:**

What was the rate of computed tomography lung cancer screening (LCS) among individuals both eligible and ineligible for screening according to the 2013 US Preventive Services Task Force recommendation?

**Findings:**

In this cross-sectional study of the 2019 Behavioral Risk Factor Surveillance System survey, the rate of LCS was 12.8% among the screening-eligible population. Of those who were screened, only 20.9% met all screening eligibility criteria (age, pack-year, and years since quit), whereas 20.1% failed to meet any criteria.

**Meaning:**

These findings suggest underuse and potential overuse of computed tomography for LCS and highlight the need to implement optimal screening strategies.

## Teaser

This cross-sectional study evaluates the rate of computed tomography (CT) lung cancer screening among patients meeting the eligibility criteria of the US Preventive Services Task Force (USPSTF).

## Introduction

Lung cancer is the second most common cancer (not including skin cancer) among adults^1^ and the leading cause of cancer-related deaths in the US.^2^ Clinical trials have demonstrated the efficacy of low-dose computed tomography (LDCT) in reducing lung cancer mortality for high-risk populations.^3,4,5^ Following the National Lung Screening Trial (NLST), in 2013 the US Preventive Services Task Force (USPSTF) recommended LDCT for lung cancer screening (LCS) for asymptomatic individuals who met 3 eligibility criteria: (1) age 55 to 80 years, (2) at least a 30 pack-year smoking history, and (3) current smokers or former smokers who had quit within the past 15 years (ie, years since quitting [YSQ] ≤15).^6^ According to recent evidence,^7^ the USPSTF updated and expanded the eligibility criteria in 2021 to (1) age 50 to 80 years and (2) at least a 20 pack-year smoking history, while retaining criteria for current smokers or former smokers with YSQ 15 years or less.^8^

The goal of LCS is early detection among asymptomatic high-risk individuals. However, screening rates among those who meet the LCS eligibility criteria have remained low, with a rate less than 20% as of 2018.^9^ Despite the slow uptake of LCS among the screening-eligible population, the use of CT for LCS among individuals who do not meet the eligibility criteria has increased over time.^10,11^ Huo et al^10^ reported the rate of CT screening for lung cancer increased from 0.8% in 2010 to 1.2% in 2015 among nonsmokers, and from 1.5% to 2.7% among low-risk smokers. The health care system would encounter a misallocation of limited resources if a large proportion of individuals who met the LCS eligibility criteria did not undergo CT, while at the same time a proportion of individuals who received CT for LCS did not meet the eligibility criteria. The latter scenario does not necessarily reflect inappropriate use of CT because physicians may have made a recommendation for screening according to their clinical judgment of individuals’ risk of lung cancer, often derived from a risk calculator.^12,13^

Several studies have used the Behavioral Risk Factor Surveillance System (BRFSS) data to estimate the rate of LCS.^14,15,16,17,18,19^ These studies mainly focused on individuals who met all 3 eligibility criteria set by the USPSTF. However, little is known about whether the population who underwent LCS met the screening eligibility criteria or the characteristics of individuals who were screened despite not meeting eligibility criteria. Using the 2019 BRFSS, this study contributes to the literature by (1) updating the rate of LCS among the eligible population, (2) characterizing the group of individuals who underwent CT for LCS by their level of compliance with the USPSTF eligibility criteria, and (3) examining factors associated with LCS among individuals who failed to meet all 3 eligibility criteria because their use of screening is least likely to be clinically justifiable. Findings from our study will help policy makers better understand the current state of health care resources allocation in the context of LCS.

## Methods

### Data Sources

We used BRFSS to identify respondents who underwent CT screening for lung cancer in 2019, and to assess whether the respondents met the eligibility criteria per USPSTF LCS recommendation. BRFSS is an annual health-related nationwide telephone survey in the US, covering approximately 400 000 adults from all 50 states, the District of Columbia, and US territories.^20^ BRFSS survey consisted of a core component, optional BRFSS modules, and state-added questions. Survey questions related to LCS were included in the optional BRFSS modules.^21^

This study was exempt for human participants review from the institutional review board at MD Anderson Cancer Center and was exempt from the need for informed consent for use of deidentified data. The study followed the Strengthening the Reporting of Observational Studies in Epidemiology (STROBE)^22^ reporting guideline for cross-sectional studies.

### Study Population

We identified 114 916 respondents who resided in the 20 states that administered the LCS module in the 2019 BRFSS and answered the LCS question.^23^ We determined whether a respondent had undergone LCS according to the respondent’s answer to the question, “In the last 12 months, did you have a CT or CAT scan?”^24^ Responses included (a) Yes, to check for lung cancer; (b) No (did not have a CT scan); (c) Had a CT scan, but for some other reason; (d) Don’t know/Not sure; and (e) Refused. We classified respondents who answered yes as individuals who underwent LCS and excluded respondents who answered don’t sure or refused (1779 respondents [1.55%]). We then excluded respondents who reported having a history of cancers other than skin cancer because they might have had a CT scan to check for lung metastasis (12 399 respondents [10.79%]). Next, we assessed whether a respondent met the screening eligibility criteria according to the USPSTF 2013 LCS recommendations. We calculated pack-year using the definition from the National Cancer Institute.^25^ Specifically, we first calculated the smoke years for former smokers as the difference between respondents’ age when they first started smoking and the age when they last smoked. For current smokers, we calculated the difference between respondents’ current age and the age when they first started smoking. We then calculated the pack-year by the following formula: (the mean number of cigarettes smoked per day × smoke years) / 20.^25^ For former smokers, we calculated YSQ as the difference between respondents’ current age and the age when they last smoked. Finally, we excluded respondents with missing values in any of the survey questions that were used to determine the screening eligibility criteria (4641 respondents [4.04%]).

**Figure 1.**
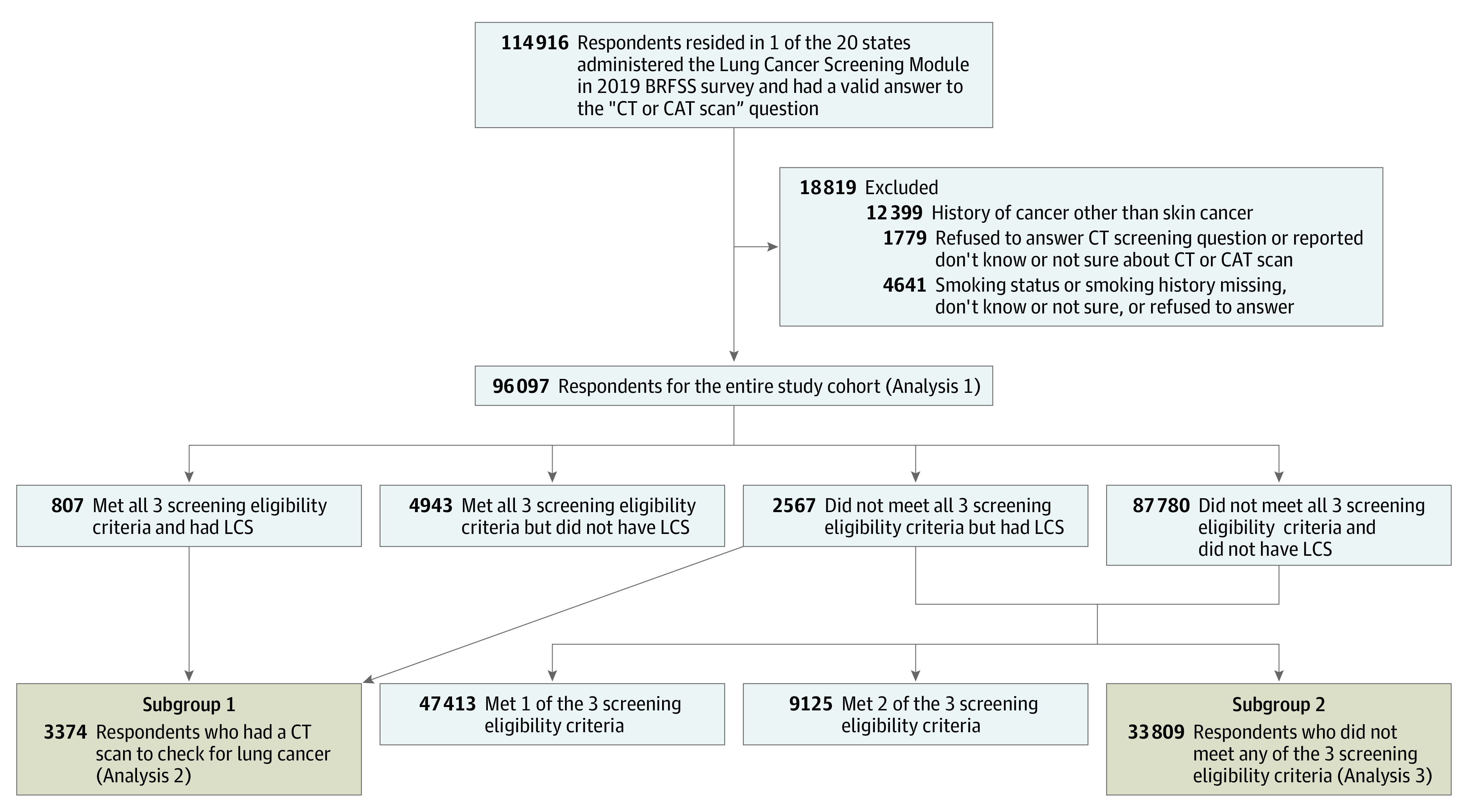
Ascertainment of Study Cohorts Screening eligibility criteria were based on the 2013 US Preventive Services Task Force (USPSTF) lung cancer screening recommendations. BRFSS indicates Behavioral Risk Factor Surveillance System; CT, computerized tomography; CAT, computerized axial tomography; LCS, lung cancer screening.

We conducted further analyses on 2 subgroups. The first subgroup contained respondents in the full study cohort who reported undergoing a CT or computed axial tomography (CAT) scan to check for lung cancer, and the second subgroup consisted of respondents who did not meet any screening eligibility criteria according to the 2013 USPSTF recommendations. See Figure 1 for cohorts ascertainment process.

### Statistical Analysis

We conducted 3 analyses. First, we used the full study cohort to estimate the overall rates of LCS, and the screening rates by state among the screening-eligible and screening-ineligible group (analysis 1). Second, we classified the respondents into 8 categories according to recommendation compliance: meeting all 3 criteria, not fulfilling 1 of the 3 criteria (age, pack-year history, or YSQ), not fulfilling 2 of the 3 criteria, and not fulfilling any criteria. We focused on the subgroup of respondents who reported having undergone LCS, calculated the proportions of these respondents in each of the 8 compliance categories, and compared the proportions according to the 2013 and 2021 USPSTF recommendations (analysis 2). Third, for the subgroup of respondents who failed to fulfill any eligibility criteria by the USPSTF 2013 recommendation, we used a logistic regression to evaluate factors associated with the use of CT for LCS (analysis 3). We conduct the same analysis for those who met all screening eligible criteria. We included age groups outside the range of the age eligibility criterion, insurance, sex, race, ethnicity, income, education, state of residence, and presence of various chronic diseases in the regression model. Our analysis used the self-reported race classification in the BRFSS: White, Black or African American, American Indian or Alaskan Native, Asian, Native Hawaiian or other Pacific Islander, and other race (other race includes races that were not specified in any of the race groups stated above). We categorized ethnicity (self-reported) as Hispanic and non-Hispanic. Race and ethnicity were assessed in this study to determine whether there were disparities among different racial and ethnic groups.

All statistical tests were 2-sided, and the statistical significance level was *P* < .05. All analyses were conducted in STATA statistical software version 15.1 (StataCorp) and had accounted for the complex survey design.^26^ Data were analyzed between October 2021 and August 2022.

## Results

We identified 96 097 respondents for the full study cohort and constructed 2 subgroups: (1) 3374 who reported having undergone a CT or CAT to check for lung cancer and (2) 33 809 who did not meet any screening eligibility criteria. Of the 96 097 respondents who answered the LCS and had complete information on their smoking history, 34 064 (weighted proportion 53.13%) were younger than 50 years, 51 536 (50.92%) were female, 88 132 (88.24%) had insurance, 84 078 (78.70%) were White, 4988 (11.20%) were Black, 4558 (8.71%) were Hispanic, and 42 665 (45.65%) had annual household income of $50 000 or greater (Table 1). Only 5750 respondents in the full study cohort met all 3 screening eligibility criteria per USPSTF 2013 recommendation (Table 1). Among them, 807 (weighted proportion 12.8%) had undergone a CT or CAT scan to check for lung cancer. Among the substantially larger group of screening-ineligible individuals, 2567 (weighted proportion 2.3%) had undergone a CT or CAT scan for LCS. These percentages correspond to the number of screening-ineligible respondents who had LCS being more than 3 times the number (2567 vs 807) in screening-eligible respondents who had LCS. Compared with the screening-ineligible population, a significantly higher proportion of the screening-eligible were insured, men, White, had an annual household income of less than $50 000, and were in the did not graduate high school or graduated high school education group. eTable 1 in the Supplement reports descriptive characteristics of the cohort stratified into 4 mutually exclusive groups: eligible, screened; eligible, unscreened; ineligible, screened; and ineligible, unscreened.

**Table 1.** Summary Statistics of Entire Population, Screening-Eligible Group, and Screening-Ineligible Group per 2013 US Preventive Services Task Force Recommendation

Characteristic	Participants, %			*P* value^b^
	Entire population^a^	Group per 2013 recommendation		
		Screening eligible	Screening ineligible	
Weighted proportion of population who underwent CT screening	2.79	12.80	2.30	<.001
Age group, y				
<50	53.13	0.00	55.65	<.001
50-54	9.09	0.00	9.52	
55-79	33.81	100.00	30.67	
≥80	3.96	0.00	4.15	
Insurance coverage status				
No insurance coverage	11.76	7.73	11.95	<.001
Have insurance coverage	88.24	92.27	88.05	
Sex				
Male	49.08	58.14	48.65	<.001
Female	50.92	41.86	51.35	
Race				
American Indian or Alaskan Native	2.11	2.48	2.10	<.001
Asian	2.19	0.36	2.28	
Black or African American	11.22	5.42	11.49	
Native Hawaiian or other Pacific Islander	0.30	0.08	0.31	
Other race^c^	2.94	1.16	3.03	
White	78.70	88.95	78.22	
Don’t know, not sure, refused to answer, no preferred race, or missing	2.52	1.56	2.57	
Ethnicity				
Hispanic	8.71	1.22	9.06	<.001
Non-Hispanic	91.29	98.78	90.94	
Annual household income, $				
<15 000	6.26	12.87	5.94	<.001
15 000 to <25 000	11.72	17.15	11.47	
25 000 to <35 000	8.31	11.67	8.15	
35 000 to <50 000	11.82	14.13	11.71	
≥50 000	45.65	29.53	46.41	
Don’t know, not sure, or missing	16.24	14.65	16.32	
Level of education				
Did not graduate high school	10.77	19.28	10.37	<.001
Graduated high school	29.85	38.78	29.43	
Attended college or technical school	32.01	30.69	32.07	
Graduated from college or technical school	27.12	11.17	27.88	
Don’t know, not sure, or missing	0.24	0.09	0.25	
Comorbidities				
Heart attack				
No	95.88	84.27	96.43	<.001
Yes	4.12	15.73	3.57	
Coronary disease				
No	96.09	86.52	96.55	<.001
Yes	3.91	13.48	3.45	
Stroke				
No	96.72	90.36	97.02	<.001
Yes	3.28	9.64	2.98	
Asthma				
No	85.26	86.11	85.22	.30
Yes	14.74	13.89	14.78	
Chronic obstructive pulmonary disease				
No	93.33	66.97	94.59	<.001
Yes	6.67	33.03	5.41	
Depression				
No	79.11	73.89	79.36	<.001
Yes	20.89	26.11	20.64	
Kidney disease				
No	97.18	93.71	97.35	<.001
Yes	2.82	6.29	2.65	
Diabetes				
No	89.49	78.59	90.01	<.001
Yes	10.51	21.41	9.99	
Smoking status				
Current	17.29	53.90	15.55	<.001
Former	23.64	46.10	22.58	
Never	59.07	0.00	61.87	
Total observations, No.				
Unweighted	96 097	5750	90 347	NA
Weighted	43 908 940	1 989 496	41 919 444	
Observations who underwent computed tomography screening, No.				
Unweighted	3374	807	2567	NA
Weighted	1 224 251	255 377	968 874	

Abbreviation: NA, not applicable.

^a^ Column percentages are weighted proportion.

^b^ *P* values were based on χ^2^ tests.

^c^ Other race includes races that were not specified in any of the race groups stated above.

**Figure 2.**
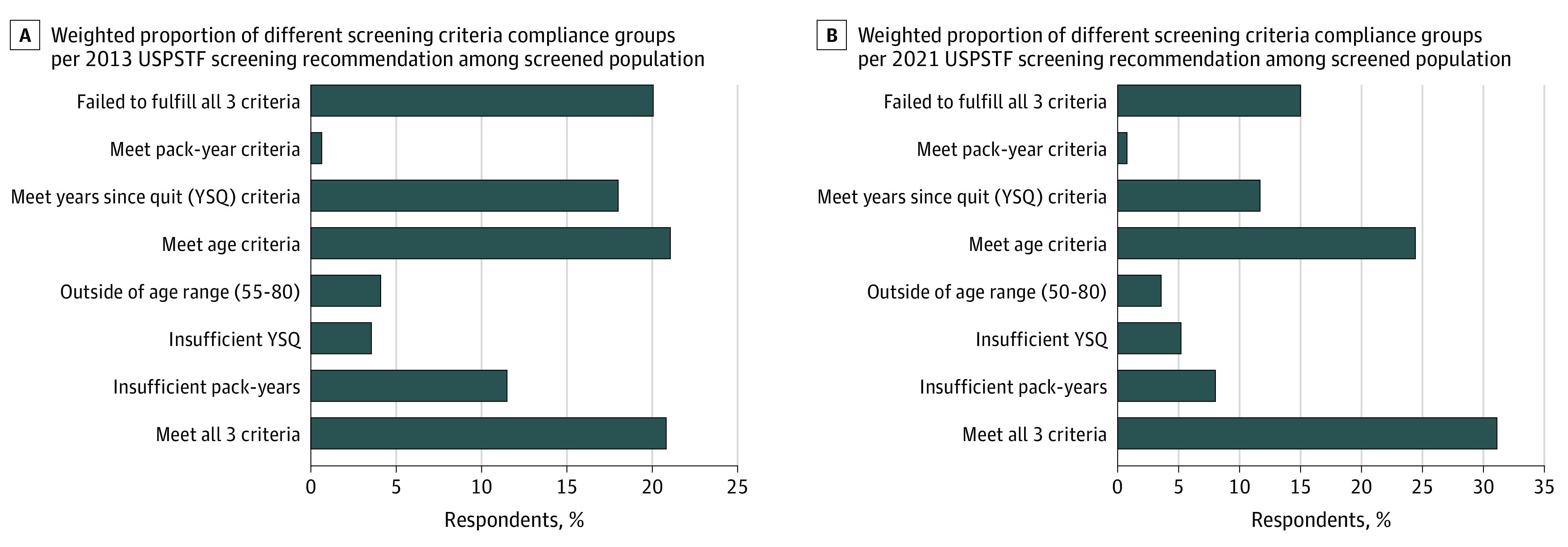
Weighted Proportion of Different Screening Compliance Groups per 2013 and 2021 US Preventive Services Task Force (USPSTF) Recommendations Among CT Screened Population

We observed wide variations in the rates of screening across states, ranging from 7.36% in Utah to 20.84% in Vermont among those who met the USPSTF 2013 eligibility criteria (Table 2). The rates among the screening-ineligible population ranged from 0.89% in Wisconsin to 5.42% in Minnesota. Among the subgroup of 3374 respondents who underwent CT for LCS, the proportion who met all 3 criteria was 807 (weighted proportion 20.9%) under the 2013 USPSTF recommendation (Figure 2A) and increased to 1101 (weighted proportion 31.1%) per the 2021 recommendation (Figure 2B), whereas the proportion who did not fulfill any eligibility criteria decreased from 20.1% to 15.0%, respectively.

**Table 2.** Proportions of Screened Population Among Eligible and Ineligible Population

State	Weighted proportion of screened population according to 2013 USPSTF recommendation, %	
	Among eligible population	Among ineligible population
Total	12.80	2.30
Arizona	11.89	2.74
Idaho	13.74	3.36
Kansas	12.54	2.22
Kentucky	16.55	2.37
Maine	13.36	3.91
Maryland	12.37	1.60
Minnesota	18.65	5.42
Missouri	14.42	1.77
Montana	16.05	1.68
Nebraska	9.73	1.96
North Carolina	9.30	2.56
North Dakota	10.93	1.29
Oklahoma	8.71	3.50
Pennsylvania	13.39	2.30
Rhode Island	17.15	2.92
South Carolina	12.57	3.51
Utah	7.36	1.21
Vermont	20.84	1.98
West Virginia	10.18	1.87
Wisconsin	13.59	0.89

Abbreviation: USPSTF, US Preventive Services Task Force.

**Figure 3.**
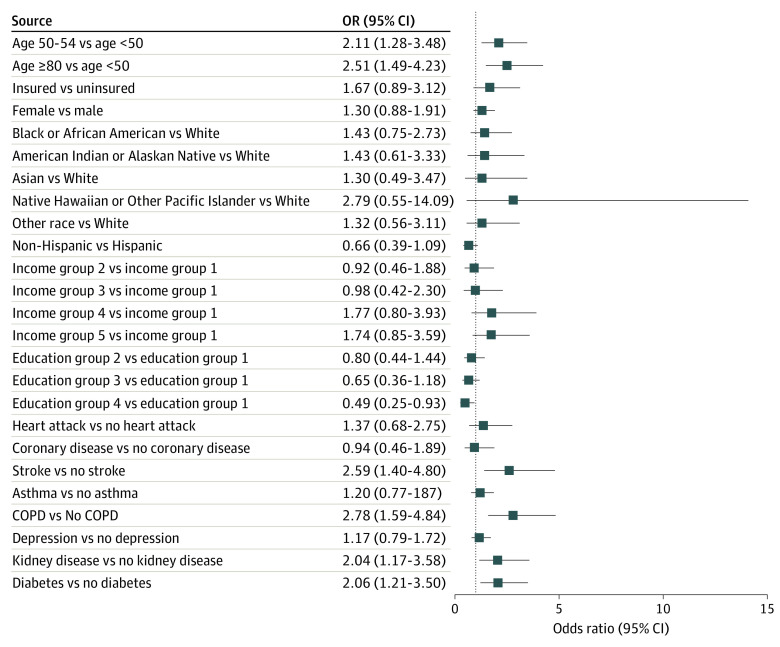
Odds Ratios (ORs) for CT Screening for Lung Cancer Among the Population Who Did Not Fulfill Any Eligibility Criteria in From the 2013 US Preventive Services Task Force Recommendation COPD indicates chronic obstructive pulmonary disease.

Among the subgroup of 33 809 respondents who failed to fulfill all 3 eligibility criteria according to the USPSTF 2013 recommendation, logistic regression shows respondents with a history of stroke (odds ratio [OR], 2.6; 95% CI, 1.4-4.8; *P* < .001), chronic obstructive pulmonary disease (COPD) (OR, 2.8; 95% CI, 1.6-4.9; *P* < .001), kidney disease (OR, 2.0; 95% CI, 1.1-3.6; *P* = .01), and diabetes (OR, 2.1; 95% CI, 1.2-3.6; *P* < .001) had significantly higher odds of LCS compared with respondents without a history of each condition (Figure 3). Among the 538 screened respondents who failed to fulfill all 3 eligible criteria, 178 had at least 1 of the comorbidities mentioned previous. If we considered their use of CT to be clinically appropriate, it would still leave 360 respondents who received LCS despite not meeting any eligibility criteria and not having any of these comorbidities. When extrapolating to the national estimate, these 360 respondents accounted for 13.7% of the screened population, representing 167 722 adults in the US. Screening for these individuals likely represents misallocation of health care resources. In addition, compared with respondents who were younger than than 50 years, those aged 50 to 54 years (OR, 2.1; 95% CI, 1.3-3.5; *P* < .001) and who were aged 80 years or older (OR, 2.4; 95% CI, 1.4-4.0; *P* < .001) had significantly higher odds of LCS. Compared with respondents who did not graduate high school, respondents who graduated from college or technical school had a low odds (OR, 0.4; 95% CI, 0.2-0.9; *P* = .02) of screening. Results of logistic regressions for the subgroups of individuals who failed all 3 eligibility criteria vs who met all 3 eligibility criteria are presented and discussed in eTable 2 in the Supplement.

## Discussion

This cross-sectional study analyzed a national survey of respondents who answered questions related to LCS in the BRFSS 2019. Our analysis has 3 main findings. First, the rate of LCS among the screening-eligible population was 12.8% according to the 2013 USPSTF recommendation and varied across states. Second, among those who reported having undergone a CT or CAT scan for LCS, only 20.9% met all 3 screening eligibility criteria in the 2013 USPSTF recommendations, whereas 20.1% of them failed to fulfill any of the 3 criteria; the percentage reduced to 15.0% according to the 2021 recommendation. Third, we identified variables associated with LCS when failing to meet any USPSTF criteria.

The low rate of LCS reported here is concerning. Although the rate has increased over time,^9^ it remains lower than 20%, even after more than 5 years since the USPSTF released its LCS recommendations in 2013 and 4 years after the Centers for Medicare & Medicaid Services began to cover LCS with low-dose CT.^27^ Consistent with other reports illustrating the state-level variation in screening utilization before 2019,^9^ our results reaffirmed that the wide variation in the uptake rate across states persisted in 2019.

The most surprising finding is the large proportion of respondents who underwent LCS yet did not meet at least 1 screening eligibility criteria. Of the 3374 respondents (representing more than 1.2 million adults in the US) who underwent a CT or CAT scan for LCS, only 20.9% met all 3 eligibility criteria. This is after excluding respondents with a history of cancer and those who had a scan for some other reason. This finding implies that 79% of individuals who underwent screening consisted of those who did not meet the screening eligibility criteria, representing close to 0.9 million adults in the US. Our findings echo the concerns expressed in prior research regarding misallocation of health care resources in LCS settings.^10,11^

It is, however, important not to label everyone who underwent CT screening but did not meet the USPSTF screening eligibility criteria as receiving inappropriate care. We analyzed the subgroup of individuals who did not meet any eligibility criteria to gain more insight on their characteristics. Among them, individuals with a history of stroke, COPD, kidney disease, and diabetes were more likely to undergo LCS, with the strongest association observed in COPD. Research has found an association of these comorbidities, especially COPD, with increased risk of lung cancer.^28,29,30,31^ These comorbidities, however, were not significantly associated with LCS among the screening-eligible individuals (eTable 2 in the Supplement), suggesting that physicians may use comorbidity profiles to make screening recommendations for those who did not meet any eligibility criteria. If the scans were reallocated from those who failed to fulfill all 3 eligible criteria and did not have comorbidities associated with lung cancer risk to people who met the screening eligibility criteria, the rate of LCS would be increased further..

A significant concern is the unnecessary financial, physical, and psychological burdens associated with LCS for low-risk individuals who fail to meet screening eligibility criteria. The false-positive rate reported in the NLST was as high as 20%.^32^ Even with the rate reduced to 5.3% according to Lung-RADS classification system, a substantial number of individuals would still be negatively affected by potential downstream events including invasive diagnostic procedures.^33^ For those who undergo invasive procedures, complication rates of these procedures observed in clinical practice settings were found to be higher than those documented in the NLST.^34^ Costs associated with complications can be as high as $56 845.^35^

Research has shown the association between insurance and LCS among the screening-eligible population.^36^ With grade B recommendation from the USPSTF, private insurance is expected to cover LDCT without cost-sharing for the screening-eligible population under the Patient Protection and Affordable Care Act.^37,38,39^ Having insurance can produce a spillover effect to the screening-ineligible population, as prior research has shown positive associations between insurance and use of low-value preventive care.^40^ In addition, clinicians who are financially motivated may promote LCS even among screening-ineligible patients. It has been reported that the downstream net revenue was $770 per screened case.^41^ Although our analysis of respondents who failed to meet any eligibility criteria found a positive association between insurance and the receipt of CT screening, the association was not significant.

The large proportion of ineligible individuals in the cohort who reported having CT screening for lung cancer could also reflect inadequacies in the USPSTF screening eligibility criteria. A necessary condition to meet the USPSTF LCS eligibility criteria is being a current or former smoker. Even with less restrictive eligibility criteria in the USPSTF 2021 recommendation, close to 70% of the screened population was considered screening ineligible, suggesting the need to explore implementation strategies for optimal use of limited resources for LCS. Although unverifiable with the BRFSS data, physicians are likely to make screening recommendations using risk prediction models regardless of patients’ age and/or smoking status. Such practice would be consistent with the third recommendation in the recent American College of Chest Physicians LCS guideline.^42^

### Limitations

This study has several limitations. First, the BRFSS did not capture all factors associated with risk of lung cancer, such as family history, symptoms (cough, hemoptysis), and exposure to hazardous chemicals such as asbestos. Second, information about LCS or smoking eligibility from the BRFSS were not complete. For the question on LCS, 1.55% of the respondents refused or reported “didn’t know/not sure.” For the smoking history questions, 4.04% of the respondents refused, reported “didn’t know/not sure”, or missed answers. Also, it may not be easy for respondents to accurately report the mean number of cigarettes smoked per day. Nevertheless, the smoking history related measures of BRFSS proves to be reliable and consistent with other national surveys.^43,44^ Third, research suggests primary care physicians and specialists may hold different opinions and perspectives of LDCT for LCS among high-risk population.^45,46^ However, we were not able to determine the type of physicians who ordered the CT scan for LCS. More research is needed to explore differences in the understanding and execution of LCS across medical professions and potential biases arisen from such differences. Fourth, the question in the BRFSS LCS module does not explicitly differentiate between conventional CT and LDCT. Therefore, it is possible that respondents who were classified as having LCS may have received conventional chest CT for other reasons. Despite the lack of specificity on LDCT in the questionnaire, the BRFSS is one of few available pieces of data to generate population-based estimates of the rate of LCS.^17^ Fifth, most states with higher lung cancer death rates were in the southeastern region of the US but were not included in the 2019 BRFSS 2019 LCS module.^47^ To obtain a more precise, clinically informative, and policy-relevant estimate of the rate of LCS at national and state level, we recommend modifying the survey question to specifically ask for the use of LDCT for LCS, and to engage states with high disease burden of lung cancer to participate in the LCS module.

## Conclusions

In this cross-sectional study of the 2019 BRFSS survey, major findings included that among individuals who underwent CT for LCS, 20.9% met the USPSTF screening eligibility criteria, and 20.1% of them did not meet any of the 3 eligibility criteria. Future research is needed to better understand the personal and clinical factors underpinning the underuse of CT screening among the screening-eligible population and its overuse among screening-ineligible individuals to optimize resources allocation for LCS.

## Notes

Supplement. **eTable 1. **Summary Statistics of Study Cohort by Eligibility and Screening Status **eTable 2. **Odds Ratio and 95% CI of Characteristics for CT Screening Among the Population Who Did Not Fulfill Any Eligibility Criteria and Who Met All Three Eligibility Criteria of the 2013 USPSTF Recommendation
