# Supplementary material for: Assessment of Uptake Appropriateness of Computed Tomography for Lung Cancer Screening According to Patients Meeting Eligibility Criteria of the US Preventive Services Task Force
Source: JAMA Netw Open. 2022 Nov 21;5(11):e2243163. doi: 10.1001/jamanetworkopen.2022.43163 (PMC9679877; doi:10.1001/jamanetworkopen.2022.43163)
Supplement: Supplement. — eTable 1. Summary Statistics of Study Cohort by Eligibility and Screening Status eTable 2. Odds Ratio and 95% CI of Characteristics for CT Screening Among the Population Who Did Not Fulfill Any Eligibility Criteria and Who Met All Three Eligibility Criteria of the 2013 USPSTF Recommendation [file jamanetwopen-e2243163-s001.pdf]

Supplemental Online Content

Liu Y, Pan IWE, Tak HJ, Vlahos I, Volk R, Shih YCT. Assessment of uptake appropriateness of computed tomography for lung cancer screening according to patients meeting eligibility criteria of the US Preventive Services Task Force. *JAMA Netw Open*. 2022;5(11):e2243163. doi:10.1001/jamanetworkopen.2022.43163

eTable 1. Summary Statistics of Study Cohort by Eligibility and Screening Status

eTable 2. Odds Ratio and 95% CI of Characteristics for CT Screening Among the Population Who Did Not Fulfill Any Eligibility Criteria and Who Met All Three Eligibility Criteria of the 2013 USPSTF Recommendation

This supplemental material has been provided by the authors to give readers additional information about their work.

© 2022 Liu Y et al. *JAMA Network Open.*

eTable 1: Summary Statistics of Study Cohort by Eligibility and Screening Status

	Eligible, screened	Eligible, unscreened	P-value		Ineligible, screened	Ineligible, unscreened	P-value		Total
Total Unweighted Observations	807	4,943			2,567	87,780			96,097
Total Weighted Observations	255,377	1,734,119			968,875	41,000,000			43,958,370
Age Group									
Age <50	0.00%	0.00%	NA		33.09%	56.18%	P < 0.01		53.13%
Age 50 to 54	0.00%	0.00%			14.81%	9.40%			9.09%
Age 55 to 79	100.00%	100.00%			45.78%	30.32%			33.81%
Age 80 +	0.00%	0.00%			6.33%	4.10%			3.96%
Insurance Coverage Status									
No Insurance Coverage	2.15%	8.55%	P < 0.01		7.48%	12.06%	P < 0.01		11.76%
Have Insurance Coverage	97.85%	91.45%			92.52%	87.94%			88.24%
Sex									
Male	56.21%	58.42%	P = 0.53		45.03%	48.74%	P = 0.07		49.08%
Female	43.79%	41.58%			54.97%	51.26%			50.92%
Race									
American Indian or Alaskan Native	1.65%	2.60%	P = 0.62		2.92%	2.08%	P = 0.69		2.11%
Asian	0.11%	0.40%			2.77%	2.27%			2.19%
Black or African American	4.76%	5.51%			12.52%	11.47%			11.22%
Native Hawaiian or Other Pacific Islander	0.02%	0.08%			0.49%	0.31%			0.30%
Other Race	0.51%	1.26%			2.50%	3.04%			2.94%
White	91.28%	88.61%			76.71%	78.25%			78.70%
Race Don’t know/Not sure/Refused to Answer/No preferred race/Missing	1.67%	1.54%			2.09%	2.58%			2.52%
Ethnicity									
Hispanic	1.91%	1.12%	P = 0.39		7.01%	9.11%	P=0.10		8.71%
Non-Hispanic	98.09%	98.88%			92.99%	90.89%			91.29%
Annual Household Income									
Annual Household Income Less than $15,000	11.05%	13.14%			9.32%	5.86%			6.26%

© 2022 Liu Y et al. *JAMA Network Open.*

Annual Household Income at least $15,000 to Less Than $25,000	16.82%	17.20%	P = 0.51		15.21%	11.38%	P < 0.01		11.72%
Annual Household Income at least $25,000 to less than $35,000	13.83%	11.35%			8.71%	8.14%			8.31%
Annual Household Income at least $35,000 to less than $50,000	15.66%	13.90%			13.24%	11.67%			11.82%
Annual Household Income $50,000 or more	25.89%	30.06%			39.96%	46.56%			45.65%
Income Don't know/Not sure/Missing	16.76%	14.33%			13.55%	16.39%			16.24%
Level of Education									
Education: Did Not Graduate High School	17.06%	19.60%	P = 0.43		15.04%	10.26%	P < 0.01		10.77%
Education: Graduated High School	36.36%	39.13%			30.08%	29.42%			29.85%
Education: Attended College or Technical School	33.74%	30.24%			36.02%	31.98%			32.01%
Education: Graduated from College or Technical School	12.84%	10.92%			18.81%	28.10%			27.12%
Education: Don't know/Not sure/Missing	0.00%	0.11%			0.05%	0.25%			0.24%
Comorbidities									
No Heart Attack	80.57%	84.81%	P = 0.07		91.56%	96.55%	P < 0.01		95.88%
Heart Attack	19.43%	15.19%			8.44%	3.45%			4.12%
No Coronary Disease	84.80%	86.77%	P = 0.38		90.98%	96.68%	P < 0.01		96.09%
Coronary Disease	15.20%	13.23%			9.02%	3.32%			3.91%
No Stroke	88.24%	90.68%	P = 0.20		91.21%	97.15%	P < 0.01		96.72%
Stroke	11.76%	9.32%			8.79%	2.85%			3.28%
No Asthma	81.76%	86.75%	P = 0.03		77.50%	85.41%	P < 0.01		85.26%
Asthma	18.24%	13.25%			22.50%	14.59%			14.74%
No COPD	48.36%	69.71%	P < 0.01		78.10%	94.98%	P < 0.01		93.33%
COPD	51.64%	30.29%			21.90%	5.02%			6.67%
No Depression	69.13%	74.59%	P = 0.06		70.84%	79.56%	P < 0.01		79.11%
Depression	30.87%	25.41%			29.16%	20.44%			20.89%
No Kidney Disease	93.16%	93.79%	P = 0.71		92.42%	97.46%	P < 0.01		97.18%
Kidney Disease	6.84%	6.21%			7.58%	2.54%			2.82%
No diabetes	76.35%	78.92%	P = 0.36		80.61%	90.23%	P < 0.01		89.49%
Diabetes	23.65%	21.08%			19.39%	9.77%			10.51%
Smoking Status									
Current Smoker	47.32%	54.87%	P = 0.03		29.04%	15.23%	P<0.01		17.29%
Former Smoker	52.68%	45.13%			31.17%	22.37%			23.64%

© 2022 Liu Y et al. *JAMA Network Open.*

Never Smoker	0.00%	0.00%			39.79%	62.40%			59.07%

Note:

* Column percentage, weighted proportion.

^ P values were based on chi-squared tests.

Among screening eligible population, insurance coverage and comorbidities of asthma, COPD, and smoking status were significantly associated with lung cancer screening. Among screening ineligible population, significant factors that associated with lung cancer screening include age, insurance, household income, levels of education, smoking status, and any comorbidities.

© 2022 Liu Y et al. *JAMA Network Open.*

eTable 2: Odds Ratio and 95% CI of Characteristics for CT Screening Among the Population Who Did Not Fulfill Any Eligibility Criteria and Who Met All Three Eligibility Criteria of the 2013 USPSTF Recommendation

		Characteristics Associated with Screening among Individuals Failed all Three Eligibility Criteria			Characteristics Associated with Screening among Individuals Met all Three Eligibility Criteria		
		**Odds Ratio and 95% Confidence Interval**	**P-value**		**Odds Ratio and 95% Confidence Interval**	**P-value**	
	Age 50 to 54 vs Age <50	2.11 [1.28, 3.48]	p < 0.01		NA	NA	
	Age 80 + vs Age <50	2.51 [1.49, 4.23]	p < 0.01		NA	NA	
	Have Insurance Coverage vs No Insurance Coverage	1.67 [0.89, 3.12]	p = 0.11		4.33 [1.89, 9.9]	p < 0.01	
	Female vs Male	1.3 [0.88, 1.91]	p = 0.19		0.96 [0.7, 1.31]	p = 0.78	
	Black vs White	1.43 [0.75, 2.73]	p = 0.28		1.1 [0.49, 2.46]	p = 0.81	
	American Indian or Alaskan Native vs White	1.43 [0.61, 3.33]	p = 0.41		0.6 [0.27, 1.37]	p = 0.23	
	Asian vs White	1.3 [0.49, 3.47]	p = 0.6		0.79 [0.08, 8.35]	p = 0.85	
	Native Hawaiian or Other Pacific Islander vs White	2.79 [0.55, 14.09]	p = 0.21		0.33 [0.03, 3.54]	p = 0.36	
	Other Race vs White	1.32 [0.56, 3.11]	p = 0.52		0.26 [0.07, 0.92]	p = 0.04	
	Race Don’t know/Not sure/Refused to Answer/No preferred race/Missing vs White	1.58 [0.63, 3.99]	p = 0.33		0.91 [0.36, 2.32]	p = 0.84	
	Non-Hispanic vs Hispanic	0.66 [0.39, 1.09]	p = 0.11		0.34 [0.11, 1.05]	p = 0.06	

© 2022 Liu Y et al. *JAMA Network Open.*

	Annual Household Income at least $15,000 to Less Than $25,000 vs Annual Household Income Less than $15,000	0.92 [0.46, 1.88]	p = 0.83		1.22 [0.77, 1.94]	p = 0.41	
	Annual Household Income at least $25,000 to less than $35,000 vs Annual Household Income Less than $15,000	0.98 [0.42, 2.3]	p = 0.99		1.65 [0.93, 2.92]	p = 0.10	
	Annual Household Income at least $35,000 to less than $50,000 vs Annual Household Income Less than $15,000	1.77 [0.8, 3.93]	p = 0.16		1.42 [0.81, 2.49]	p = 0.23	
	Annual Household Income $50,000 or more vs Annual Household Income Less than $15,000	1.74 [0.85, 3.59]	p = 0.13		1.16 [0.74, 1.84]	p = 0.52	
	Income Don't know/Not sure/Missing vs Annual Household Income Less than $15,000	0.63 [0.32, 1.26]	p = 0.20		1.44 [0.85, 2.42]	p = 0.20	
	Education: Graduated High School vs Education: Did Not Graduate High School	0.8 [0.44, 1.44]	p = 0.45		1.18 [0.77, 1.8]	p = 0.46	
	Education: Attended College or Technical School vs Education: Did Not Graduate High School	0.65 [0.36, 1.18]	p = 0.16		1.5 [0.97, 2.34]	p = 0.07	
	Education: Graduated from College or Technical School vs Education: Did Not Graduate High School	0.49 [0.25, 0.93]	p = 0.03		1.53 [0.95, 2.48]	p = 0.08	
	Heart Attack vs No Heart Attack	1.37 [0.68, 2.75]	p = 0.38		1.19 [0.81, 1.75]	p = 0.40	
	Coronary Disease vs No Coronary Disease	0.94 [0.46, 1.89]	p = 0.86		0.83 [0.54, 1.28]	p = 0.40	
	Stroke vs No Stroke	2.59 [1.4, 4.8]	p < 0.01		1.14 [0.75, 1.74]	p = 0.53	
	Asthma vs No Asthma	1.2 [0.77, 1.87]	p = 0.41		1.02 [0.7, 1.48]	p = 0.92	
	COPD vs No COPD	2.78 [1.59, 4.84]	p < 0.01		2.58 [1.87, 3.56]	p < 0.01	
	Depression vs No Depression	1.17 [0.79, 1.72]	p = 0.44		1.13 [0.84, 1.52]	p = 0.43	

© 2022 Liu Y et al. *JAMA Network Open.*

	Kidney Disease vs No Kidney Disease	2.04 [1.17, 3.58]	p = 0.01		0.9 [0.52, 1.57]	p = 0.72	
	Diabetes vs No Diabetes	2.06 [1.21, 3.5]	p = 0.01		1.06 [0.75, 1.49]	p = 0.75	

Note:

^ P values were based on chi-squared tests.

Insurance coverage was a statistically significant factor associated with LCS among screening eligible population, but not the population who violated all three eligibility criteria. If individuals were eligible for lung cancer screening, comorbidities of stroke, kidney disease, and diabetes were no longer statically significant factors associated with LCS like those failed all three eligibility criteria.

© 2022 Liu Y et al. *JAMA Network Open.*
